# Coronary microvascular injury in myocardial infarction: perception and knowledge for mitochondrial quality control

**DOI:** 10.7150/thno.60143

**Published:** 2021-05-03

**Authors:** Xing Chang, Amanda Lochner, Hsueh-Hsiao Wang, Shuyi Wang, Hang Zhu, Jun Ren, Hao Zhou

**Affiliations:** 1 Chinese PLA General Hospital, Medical School of Chinese PLA, Beijing 100853, China.; 2 Guang'anmen Hospital of Chinese Academy of Traditional Chinese Medicine, Beijing, China.; 3 Department of Biomedical Sciences, Faculty of Health Sciences, University of Stellenbosch, Stellenbosch, South Africa.; 4 Department of Medicine, Mackay Medical College, New Taipei City, 252, Taiwan.; 5 University of Wyoming College of Health Sciences, Laramie, WY 82071, USA.; 6 Shanghai University School of Medicine, Shanghai 200044, China.

**Keywords:** coronary microvasculature, ECs, myocardial infarction, mitochondrial quality control

## Abstract

Endothelial cells (ECs) constitute the innermost layer in all blood vessels to maintain the structural integrity and microcirculation function for coronary microvasculature. Impaired endothelial function is demonstrated in various cardiovascular diseases including myocardial infarction (MI), which is featured by reduced myocardial blood flow as a result of epicardial coronary obstruction, thrombogenesis, and inflammation. In this context, understanding the cellular and molecular mechanisms governing the function of coronary ECs is essential for the early diagnosis and optimal treatment of MI. Although ECs contain relatively fewer mitochondria compared with cardiomyocytes, they function as key sensors of environmental and cellular stress, in the regulation of EC viability, structural integrity and function. Mitochondrial quality control (MQC) machineries respond to a broad array of stress stimuli to regulate fission, fusion, mitophagy and biogenesis in mitochondria. Impaired MQC is a cardinal feature of EC injury and dysfunction. Hence, medications modulating MQC mechanisms are considered as promising novel therapeutic options in MI. Here in this review, we provide updated insights into the key role of MQC mechanisms in coronary ECs and microvascular dysfunction in MI. We also discussed the option of MQC as a novel therapeutic target to delay, reverse or repair coronary microvascular damage in MI. Contemporary available MQC-targeted therapies with potential clinical benefits to alleviate coronary microvascular injury during MI are also summarized.

## Introduction

**Figure 1:**
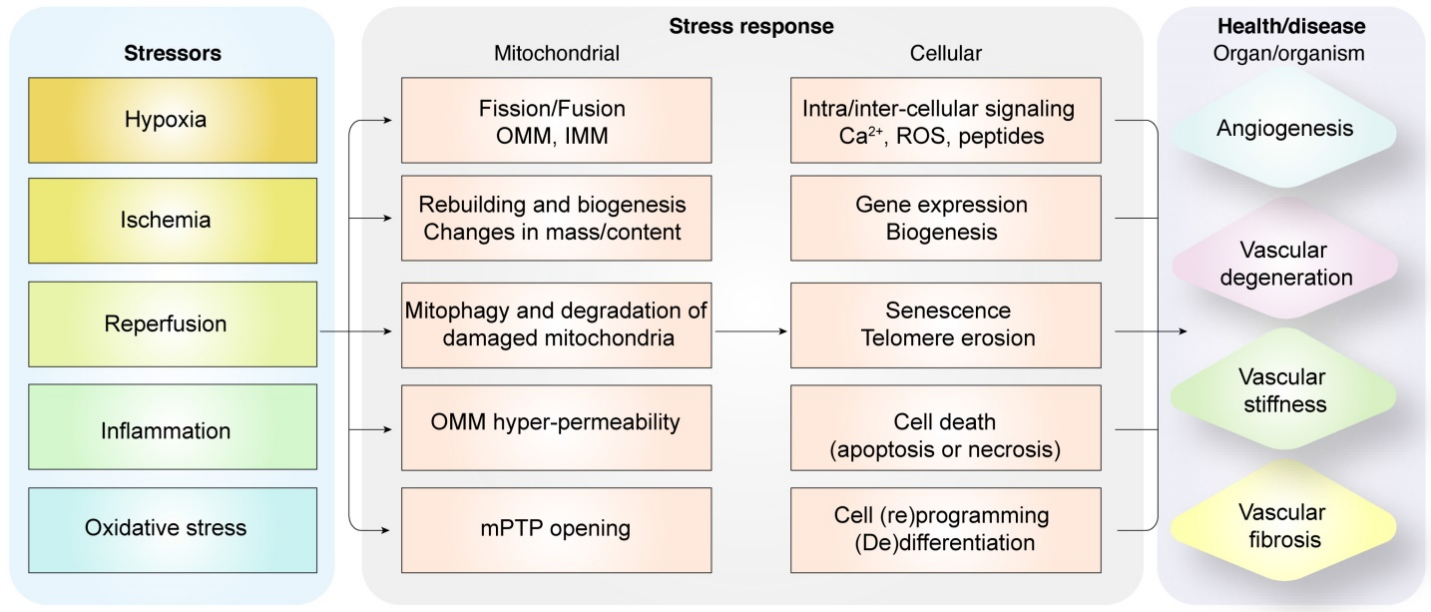
** Diagrammatic representation shows the relationship between pathological alterations in vasculature during myocardial infarction and mitochondrial quality control mechanisms.** Intrinsic and extrinsic stress signals such as hypoxia, ischemia, reperfusion, inflammation or oxidative stress activate mitochondrial fission or fusion. Mitophagy promotes degradation of damaged mitochondria. Mitochondrial biogenesis regulates synthesis of mitochondrial DNA, proteins, and lipids to restore optimal mitochondrial mass. Outer mitochondrial membrane permeabilization (OMM) is induced and mitochondrial permeability transition pore (mPTP) opening is enhanced when mitochondrial damage becomes excessive. OMM promotes cellular apoptosis, whereas, mPTP opening induces necrosis or necroptosis. Dysregulated mitochondrial quality control (MQC) mechanisms induce aberrant calcium signaling, excessive ROS, changes in gene expression of proteins related to bioenergetics, senescence, telomere erosion, metabolic reprogramming, (de)differentiation, or cell death (apoptosis or necrosis) in the coronary microvascular endothelial cells. These changes in endothelial cells, coronary arteries or cardiac microcirculation induce vascular degeneration, stiffness, or fibrosis and reduce angiogenesis.

Coronary microcirculation is usually present in vessels with diameters ranging 200-400 μm and is invisible under coronary angiography. Unlike macrocirculation which is mainly carried by epicardial arteries (diameter > 400 µm), coronary microvasculature consists of small arteries (diameter < 400 µm), arterioles (diameter < 100 µm) and capillaries (diameter < 10 µm). The function of macrocirculation encompasses the delivery of fresh blood, oxygen and nutrients as well as the removal of carbon dioxide. Coronary microcirculation is widely employed by human body to control of distribution of blood flow, with an important role in the regulation of vascular resistance. Endothelial cells (ECs), vascular smooth muscle cell (VSMC) and pericytes are the main components of coronary microvasculature. The coronary microvasculature regulatory control mechanisms were recently systematically reviewed by Heusch and colleagues 1. The interior surface of coronary microcirculation is lined by a monolayer of ECs and acts as a conduit for transporting blood and nutrients. ECs also serve as a defensive barrier against infiltration of micro-organisms, immune cells, and coagulation components, thereby reducing risk of thrombosis. Vascular endothelium is a dynamic endocrine organ consisting of multiple populations of ECs with different architectural and functional properties that synergistically promotes an anti-thrombotic and anti-inflammatory environment and maintains the tissue perfusion. The coronary microvascular tone is regulated by the balance between vasodilators such as bradykinin and nitric oxide (NO) and vasoconstrictors such as endothelin, both of which are released by ECs. Endothelial dysfunction and/or coronary microvascular impairment due to inflammation or oxidative stress is the main cause of several cardiovascular disorders 2. For example, myocardial infarction (MI) is caused by coronary thrombosis (coronary microvascular spasm or occlusion) **(Figure 1)**. In the heart, ECs and cardiomyocytes form crosstalk with each other through the release of several cell signaling transmitters 3. However, excessive EC-cardiomyocyte signaling exchange may prompt a vicious cycle that causes cardiomyocyte injury and death.

**Figure 2:**
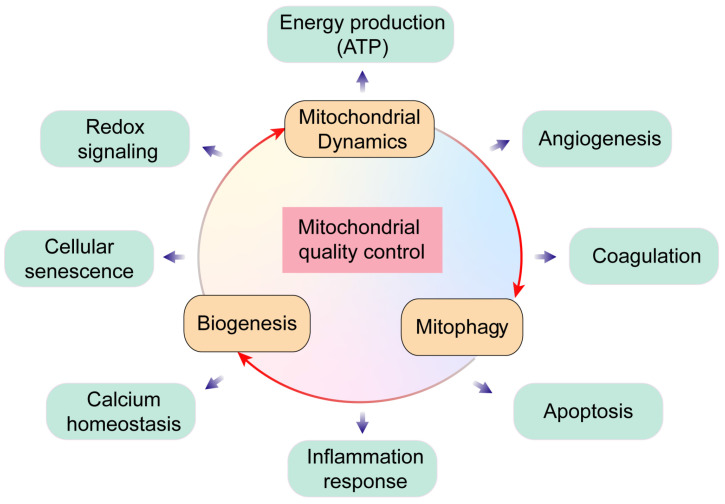
** Overview of mitochondrial quality control.** Mitochondria regulate several key biological processes in the endothelial cells including energy production, redox signaling, cellular senescence, calcium homeostasis, angiogenesis, coagulation, apoptosis and inflammation response.

Mitochondria are cellular powerhouses that produce ATP through oxidative phosphorylation. The relative mitochondrial mass compared to total cytoplasmic volume is 2-6% in ECs and ~32% in cardiomyocytes. Moreover, ECs are more dependent on glycolysis as energy source and are less dependent on oxidative phosphorylation. ECs with acquired mitochondrial abnormalities demonstrate a cancer-like phenotype including increased pyruvate dehydrogenase kinase (PDK) and pyruvate kinase muscle isoform 2 (PKM2) levels and activity associated with uncoupled glycolysis 4. Therefore, ECs would require much less oxygen from the coronary microcirculation, thereby ensuring sufficient oxygen supply to cardiomyocytes. In addition to the pseudo-hypoxic state, ECs activate the pentose phosphate pathway, a major source of cellular reducing power in the form of NADPH 5. The ECs utilize pentose phosphate pathway as the predominant anti-oxidative mechanism to protect ECs from oxidative damage and maintain cellular redox balance. In addition to metabolic switch capacity, mitochondria modulate their structure, function, and mass to meet the metabolic needs of the heart through mitochondrial quality control (MQC) mechanisms including fission, fusion, mitophagy, and biogenesis. MQC machinery plays a critical role in cardiac health and disease, while pathological changes in MQC may trigger onset and development of cardiovascular disorders including MI **(Figure 2)**.

In this review, we will first describe unique features of healthy coronary microvasculature and currently perceived pathological mechanisms that damage coronary microcirculation in MI. Then, we will highlight the distinct roles of various MQC mechanisms including fission, fusion, mitophagy, and biogenesis in coronary microvascular homeostasis under normal physiological and pathophysiological conditions such as MI. In the end, we will summarize mitochondria- and endothelium-targeted pharmacological agents with therapeutic potentials for MI.

## Pathology of coronary microvascular ECs damage in MI

### Clinical features of coronary microvascular damage in MI

MI is caused by sudden occlusion of the coronary arteries that results in reduced blood supply to the myocardium and ischemic damage to the heart. Rapid restoration of coronary flow to the ischemic myocardium (reperfusion) is the primary therapeutic strategy for MI. However, successful recanalization to restore blood flow and oxygen supply to the myocardium is associated with increased risk of reperfusion injury. Therefore, coronary microvascular injury in MI results in myocardial damage because of both ischemic and reperfusion injuries. After 180 minutes of coronary occlusion, cardiomyocytes in the ischemic myocardium show intracellular edema, swollen mitochondria with amorphous dense bodies, absence of glycogen stores, and numerous breaks in the sarcolemmal membrane 6. These alterations suggest that ischemia-mediated myocardial damage is mainly due to a transient shortage of oxygen and nutrients as well as then accumulation of breakdown products.

Ultrastructural changes in the ECs within ischemic myocardium are less prominent compared with those noted in cardiomyocytes during ischemia. The most prominent change in ECs during early ischemia is the loss of pinocytotic vesicles 7. As ischemia persists, ECs exhibit localized areas of swelling and bleb formation on the intraluminal surface, gaps between adjacent ECs, and occasional foci of hemorrhage 8. However, these changes in ECs can only be observed in 40% of the microvessels mainly localized in the sub-endocardial zones that contain irreversibly damaged cardiomyocytes after 180 minutes of coronary occlusion. This suggests that ECs in the coronary microvasculature are less prone to ischemic insult compared with cardiomyocytes and coronary microvascular damage in ECs lags behind cardiomyocyte injury 9. Although the duration of ischemia determines the extent of myocardial damage, infarcted size is also defined by the number and localization of occluded coronary arteries.

Percutaneous coronary intervention (PCI) and coronary artery bypass surgery (CABG) are the main streams treatments currently available to revascularize the occluded coronary arteries. However, more than 50% of patients with coronary microvascular damage receiving PCI or CABG displayed a no-reflow phenomenon due to inadequate myocardial reperfusion 10. In contrast to ischemic injury, both coronary microvascular dysfunction and endothelial cell death contribute to cardiac reperfusion injury 11, 12. Nonetheless, the precise molecular mechanisms underlying coronary microcirculation reperfusion injury are not well studied compared to the perceived knowledge of ischemic injury 13.

The signs of the no-reflow phenomenon despite open epicardial coronary artery in MI patients include decreased ventricular function, lower ejection fraction, and poorer clinical outcomes. Therefore, reperfusion therapeutic strategy must take into consideration to restore the functional status of myocardial microvasculature in addition to epicardial coronary artery patency 14.

During reperfusion, capillary lumen in the coronary ischemic zone is completely filled with endothelial protrusions and membrane-bound bodies including degranulated platelets 6. Moreover, Heusch and coworkers 15, 16 reported accumulation of atherothrombotic debris and soluble substances (such as serotonin, thromboxane B2, and TNFα), released from the ruptured atherosclerotic plaque in epicardial coronary artery, all of which with a pivotal role in perturbing microvascular perfusion during myocardial infarction 17.

Unlike coronary microvascular ECs, cardiomyocytes exhibit intracellular edema, relaxed myofibrils, swollen mitochondria with fractured cristae, and nuclear changes during both reperfusion and ischemic injuries. Prolonged reperfusion causes significant changes in both cardiomyocytes and coronary microcirculation. Following 3.5 h of reperfusion, myocardium displays a diffused necrosis band blended with hyper-contracted sarcomeres 18. Post-ischemic myocardium exhibits massive intravascular accumulation of neutrophils. In areas of impaired flow, a 20-fold increase in neutrophils and red blood cell stasis is observed due to plugging of capillaries by neutrophils 19. Moreover, extravascular red blood cells are frequently observed in gaps within the endothelial lining 19. In canine MI model, no-reflow zone was nearly three times greater 3.5 h later in comparison with the no-reflow zone after 2 mins of reperfusion 19. Similarly, in a rabbit MI model, the size of no-reflow zone was nearly three-fold larger after 2 h reperfusion compared to the no-reflow zone after 30 min reperfusion 20. Moreover, the rise in the size of no-reflow zone limited myocardial blood flow in the area of risk to ~44% of the baseline flow observed prior to the initiation of myocardial ischemia 21. These data favor a time-dependent worsening of coronary microvascular damage during reperfusion injury coinciding with cardiac dysfunction.

In addition to irreversible damage evoked by cardiac reperfusion injury, recognition of the role for myocardial stunning and hibernation has greatly broadened our understanding of myocardial ischemia/reperfusion injury. Myocardial stunning refers to reversible, yet slowly recovering, contractile dysfunction which follows the brief periods of myocardial ischemia. At the molecular levels, stunned myocardium following MI is not considered a cardioprotective tune-down of contractile force, but rather a core feature of post-ischemic injury. The mechanisms of myocardial stunning have been elegantly and comprehensively addressed by Bolli and Marbán 22, 23 decades ago and were carefully recapitulated by Heusch 24. Abnormal calcium signal and excessive oxidative stress have been identified as the potential mediators of myocardial stunning 22-24. In brief, ROS formation is enhanced as a result of injured mitochondrial function and activated NADPH oxidase 25. Besides, myocardial ischemia/reperfusion also induces calcium overload through increased sodium-proton exchange, reverse mode sodium-calcium exchange, and defective sarcoplasmic reticulum 26. More importantly, elevated cytoplasmic calcium load turns on NADPH oxidase to foster mitochondrial ROS generation and, in turn, ROS impairs sarcoplasmic reticulum function 27. Finally, the interplay between calcium and oxidative stress further promotes oxidative modification and proteolysis of myofibrillar proteins 28, resulting in blunted calcium responsiveness in cardiomyocytes. Short-term myocardial hibernation is characterized by reduced regional contractile function and blood flow, which both recover after reperfusion or revascularization 29. In fact, myocardial hibernation is involved in the activation of adaptive responses to sustain myocardial viability with reduced blood flow. Unlike myocardial stunning, the precise molecular basis underneath myocardial hibernation remains unclear. Abnormal calcium homeostasis 30 and defective endogenous nitric oxide 31 are perceived possible culprit factors for the occurrence of myocardial hibernation.

Altogether, MI involves irreversible and/or reversible ischemic damage and reperfusion injury. Although cardiomyocytes are deemed the primary targets for cardioprotection, damage in coronary microvascular ECs also exacerbates MI. However, molecular machineries underneath coronary microvascular damage are not well understood and require in-depth scrutiny given its prevalent role in the pathophysiology of MI.

### Oxidative stress in ECs

The basic function of cardiac coronary circulation is to optimize the delivery of oxygen and nutrients to cardiomyocytes and regulate contractile function in particular relaxation. Oxygen consumption by ECs is significantly lower when compared to that of cardiomyocytes. Therefore, greater percentage of oxygen in the blood is available to perivascular cardiomyocytes. Reactive oxygen species (ROS) are by-products of metabolism which function as secondary messengers to transmit physiological signals 32, 33. However, excessive ROS production compromises intracellular components such as phospholipids, proteins, and DNA. MI is accompanied by an imbalance between ROS production and detoxification by antioxidant enzymes. ECs are sensitive to hypoxia, especially evident during hypoxic coronary vasoconstriction. Mitochondrial complexes I and III are primary sources of ROS generation under pathological conditions. Furthermore, nicotinamide adenine dinucleotide phosphate oxidases (NOXs) are major enzymes to generate mitochondrial ROS in ECs. ECs express four different NOX isoforms-superoxide-generating NOX1, NOX2, and NOX5 as well as hydrogen peroxide-generating NOX4 34. The main downstream targets of NOX-derived ROS in the ECs include NF-κB, activated protein-1 (AP1), hypoxia-inducible factor-1 (HIF-1), p53, p38, c-Jun N-terminal kinase, and Src kinases 35. NOX-derived ROS promotes coronary microvascular damage, which then causes aberrant vasoconstriction, apoptosis, inflammation, fibrosis, hypertrophy, and aging 36. The upregulation of NOX enzymes during hypoxia increases ROS levels in the ECs and promotes angiotensin II type -1 (AT-1) receptor-mediated vasoconstriction 37. Although hypoxia-related coronary contraction is an adaptive response to accelerate blood flow under normal conditions, narrowed coronary artery such as in MI fails to deliver sufficient oxygen and glucose to cardiomyocytes. Moreover, angiotensin II stimulates endothelin-1 (ET-1), which induces cardiac hypertrophy and fibrosis 38. This finding validates a crosstalk between oxidative stress-related changes in the ECs and cardiac hypertrophy.

Coronary artery spasms are also provoked by blunted endothelium-dependent vasorelaxation, predominantly mediated by NO. The endothelium-derived NO diffuses across cellular membranes, entering into vascular smooth muscle cells (VSMCs), and increases cyclic guanosine monophosphate (cGMP) levels through soluble guanylate cyclase. Increased cGMP levels help to decrease intracellular calcium levels, en route to vasodilation. NOX-derived ROS oxidize NO to peroxynitrite, a highly reactive and vasoconstrictive ROS species to reduce the bioavailability of NO. Besides, upregulation of NOX-mediated redox signaling also disrupts endothelial barrier function 39. This suggests a link between oxidative stress and endothelial barrier integrity.

Dyslipidemia, a condition with high circulating levels of low-density lipoprotein (LDL) and low levels of high-density lipoprotein (HDL), has been considered an underlying cause of coronary artery disease or MI. Interestingly, high LDL levels upregulate NOX activity, which promotes ROS accumulation and senescence of endothelial progenitor cells 40.

### Inflammation in ECs

Coronary microvascular ECs are both active participants and regulators of inflammation in MI. ECs possess an anti-inflammatory role in the cardiovascular system under normal physiological conditions to repel neutrophils in circulation. However, in response to cardiac tissue injury, especially myocardial ischemia, ECs upregulate leukocyte-adhesion molecules and attract neutrophils to remove necrotic tissue in infarcted zones 41. In a canine model of acute myocardial ischemia injury, neutrophil accumulation occurs within 3-6 h upon activation of ECs, and peaks at around 48 h after coronary artery occlusion 42. Following reflow of fresh blood into the ischemic myocardium, rapid accumulation and initiation of neutrophil infiltration occurs within 3 minutes and peaks within 2 to 3 h 43. Neutrophil adhesion triggers the release of ROS and a positive feedback loop to further activate host inflammatory response during their transmigration into the endothelium 44. In non-inflamed cardiac tissues, ECs do not express adhesion molecules or thrombogenic factors on their surface. However, once damaged, ECs release vascular endothelial growth factor (VEGF) and stromal-derived factor 1 (SDF-1), which recruit endothelial progenitor cells (EPCs) to repair and regenerate blood vessels by promoting proliferation, differentiation, and angiogenesis of ECs 45. Damaged ECs also upregulate several adhesion molecules such as intercellular adhesion molecule 1 (ICAM1) and vascular cell adhesion molecule 1 (VCAM1), which attract pro-inflammatory cells such as neutrophils and macrophages 46. These immune cells secrete pro-inflammatory cytokines such as interleukin 6 (IL-6), tumor necrosis factor α (TNFα), and monocyte chemo-attractant protein 1 (MCP1), which induce expression of cyclo-oxygenases (COXs) in various tissues and cells, including ECs 47. Elevated levels of pro-inflammatory factors, especially COX, are tightly associated with vasoconstriction. Shreeniwas and coworkers reported that hypoxia enhances adherence of leukocytes to the cultured monolayers of human ECs 48. In addition, ample pro-inflammatory factors such as IL-6, TNFα, and MCP1 directly and indirectly trigger endothelial dysfunction and microvascular damage. These pro-inflammatory factors prompt synthesis of prostaglandins (PGs) including prostaglandin E2 (PGE2) 49. Elevated PGE2 levels and activity promotes platelet activation and adhesion to the damaged ECs, thereby further limiting blood flow in the coronary microcirculation 50.

Higher P-selectin expression in activated platelets facilitates adhesion of circulating monocytes to the walls of blood vessels 51. The damaged ECs express CD40, which interacts with CD40 ligand (CD40L) and promotes secretion of von Willebrand factor (vWF) from the endothelial Weibeo-Palade bodies 52. Moreover, interaction between endothelium and platelets through CD40 and CD40L triggers activation of macrophages and T-cells on the vessel wall and accelerates the inflammatory response 53.

Elevated ROS levels in the ECs from MI patients significantly increased levels of oxidized low-density lipoprotein (oxLDL). Circulating ox-LDL is primarily taken up by macrophages, which then migrate through damaged endothelium into sub-endothelial connective tissues. Next, macrophages differentiate into foam cells, which are wrapped by smooth muscle cells to trigger atherosclerotic plaque formation 54. In this context, EC dysfunction and EC-mediated ox-LDL generation are both involved in macrophage activation and atherosclerotic plaque formation. Tian and associates reported that endothelium-specific anti-inflammatory therapies such as nicorandil or shexiang tongxin dropping pills enhanced myocardial blood flow and cardiac function in patients with coronary microvascular diseases 55. Furthermore, drugs that reduce endothelial inflammation attenuate MI-related cardiac damage 56. These results highlight that inflammation promotes EC damage during MI.

## Coagulation

**Figure 3:**
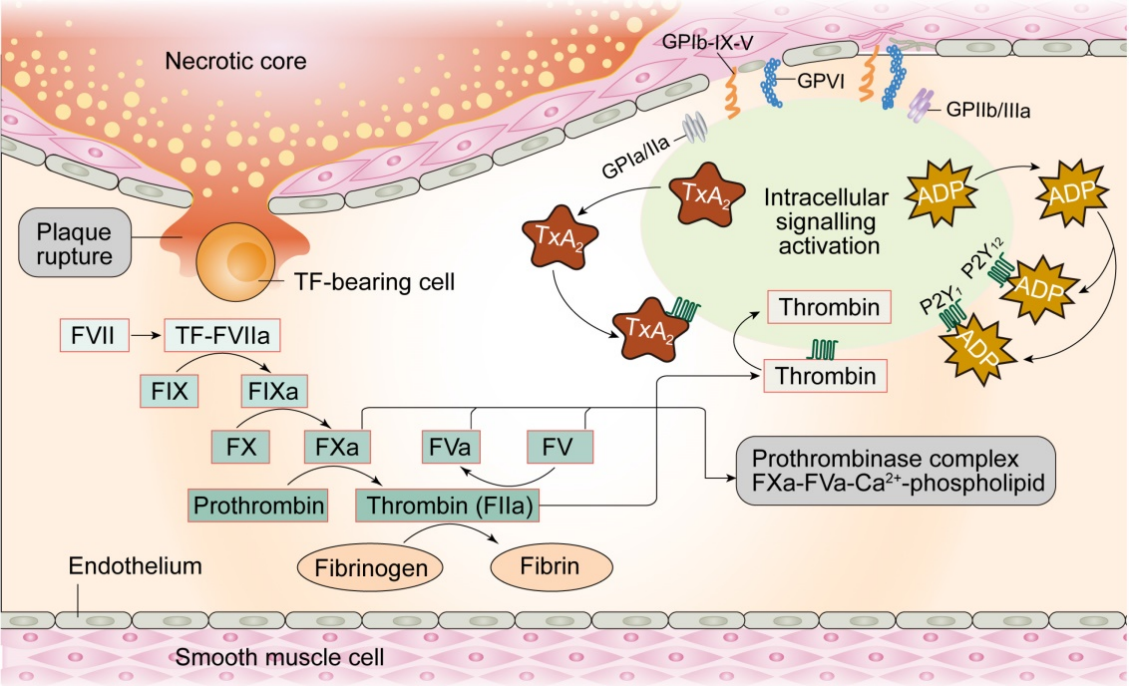
The coagulation process in endothelial cells.

Under physiological conditions, the glycocalyx layer made up of proteoglycans and glycoproteins acts as an anti-coagulation barrier between ECs and blood. The glycocalyx regulates permeability of the ECs and inhibits platelets from adhering to ECs given their negative charge **(Figure 3)**. Moreover, prostacyclins (a product of arachidonic acid metabolism in endothelial cells) with vasodilating property, inhibit platelet aggregation by elevating cAMP levels in the platelets. Furthermore, NO produced by ECs promotes increased synthesis of GMP and reduces intracellular calcium levels, GPIIb/IIIa platelet receptor transformation, and binding of integrin to fibrinogen in the platelets, thereby inhibiting their activation. Tissue factor pathway inhibitors (TFPI) located on the surface of ECs suppress the initiation of coagulation through interrupting the generation of factor VIIa-tissue-factor complexes. ECs also express thrombomodulin and specific proteoglycans to activate protein C and thus inhibit the coagulation cascade by limiting proteolysis of factors VIIIa (FVIIIa) and Va (FVa). Once thrombin is formed, ECs release tissue plasminogen activator (tPA) to break down blood clots and restore blood flow.

Considering the role of intact endothelium acts as a natural barrier against thrombogenesis, damaged ECs stimulate thrombus formation. Physical loss of endothelium exposes the sub-endothelial extracellular matrix and promotes adhesion of platelets, release of tissue factors, and local depletion of PGI2 and plasminogen activators. Thrombosis initiation can occur courtesy of perturbations in the dynamic balance between pro- and anti-thrombotic activities in the endothelium, independent of denuded or physically disrupted endothelium 57. Under pathological conditions, dysfunctional ECs upregulate pro-coagulant factors such as platelet adhesion molecules, tissue factors, plasminogen activator inhibitor-1 and downregulate anti-coagulant factors such as thrombomodulin, PGI2, and t-PA 58, creating a pro-thrombotic microenvironment. For example, damaged ECs produce TXA2, which turns on platelet activation, adhesion and aggregation onto the vascular walls 59. Activated ECs express ADAM-15, a transmembrane cell-surface protein and member of the ADAM (a disintegrin and metalloproteinase) family, prior to its binding and activation of platelets through the GIIb/IIIa receptor in the face of MI 60. Taken together, endothelium damage-mediated platelet activation followed by coronary artery thrombosis are the major perceived pathogenic mechanisms underscoring MI.

Several therapies have surfaced in MI targeting the complex interactions between platelets and ECs. The primary therapies to prevent thrombosis and reduce the risk of acute MI include classical anti-thrombotic agents such as aspirin and clopidogrel as well as novel more potent drugs such as prasugrel and ticagrelor 61. Aspirin suppresses platelet activation by inactivating cyclooxygenase-1 (COX-1), thus retarding formation of thromboxane A2 (TXA2), a potent platelet agonist and vasoconstrictor. In addition, oxidative stress and inflammation in ECs could be attenuated by aspirin 62. Supplementation of aspirin is also capable of stimulating the endothelial NO-GMP cascade to favor microvascular relaxation 63. The integrity of endothelial gap junctions could be sustained by aspirin, an effect that is followed by an inhibition of inflammatory cells infiltration 64.

Similar to aspirin, ticagrelor is a novel therapeutic reagent with proven clinical benefits compared with the classical anti-thrombotic medications 65. Ticagrelor interrupts adenosine reuptake in erythrocytes and improves cardiac blood flow in a canine model of coronary blood flow 66. Ticagrelor also promotes ATP release from red blood cells and enhances discharge of EC-related vasodilatory factors such as prostacyclin and NO into microcirculation 67. Furthermore, supplementation of ticagrelor improves brachial artery vascular tone in acute coronary syndrome (ACS) patients by enhancing nitroglycerin-mediated dilation 68. Bonello *et al*. reported that ticagrelor treatment increased the numbers of circulating EPCs in patients with MI 69. These results convincingly support that drug-induced endothelial protection may prevent thrombosis and is a plausible therapeutic approach for MI patients.

## The circulating endothelial compartment

During MI, damaged ECs shed from the micro-vessel wall and enter the circulation. MI patients show ~4.5 fold higher levels of circulating ECs (CECs) compared with healthy individuals 70. Furthermore, detachment of ECs release endothelial-derived microvesicles or microparticles (EMPs) 71. Thus, CECs and EMPs are deemed potential biomarkers of acute/chronic endothelial dysfunction in cardiovascular diseases, especially MI. EMPs derived from remodeling of the plasma membrane and externalization of phosphatidylserine are shed as submicrometric vesicles from stressed or damaged ECs 72. Moreover, apoptotic and/or necrotic CECs promote microvascular inflammation response by interacting with the healthy endothelium. EMPs are a potential source of thrombus formation due to pro-coagulation factors within EMPs such as phosphatidylserine and tissue factor (TF) 73.

Compared with the control group, total numbers of CECs are significantly elevated in patients with unstable angina in the absence of necrotic cardiomyocytes (as indicated by troponin or creatine kinase MB-fraction assays) 74. Furthermore, when compared to healthy volunteers, MI patients with occlusion of left anterior descending artery (LAD) express significantly higher levels of CD31^+^/CD42^-^ EMPs comparable to troponin T levels, although such effects did not correlate with the estimated CK-MB values 75. Numbers of CD31^+^/CD42^-^ EMPs correlate well with ischemic area at risk (MaR), but not infarct size (IS) in patients with LAD infarctions, as evaluated using magnetic resonance imaging 75. Increased numbers of CECs are associated with a higher prevalence of adverse cardiac events and cardiac remodeling in MI patients 76. In MI patients receiving anti-thrombotic therapies such as aspirin and clopidogrel, reduced levels of CECs correlate with improved left ventricular function 77. However, increased numbers of CECs are not associated with classical markers for myocardial necrosis such as creatine kinase or troponin in MI patients 77. Therefore, increased numbers of CECs should not be used as a marker for cardiomyocyte damage, but rather represent an early sign of active peri-plaque rupture that eventually leads to acute athero-thrombotic occlusion of the entire afflicted vessel and ultimate MI.

### Pericytes

Pericytes are vascular mural cells embedded within the microvascular basement membrane, enwrapping capillaries, venules and terminal arterioles 78. Coronary ECs maturation and stability are under the control of pericytes, suggesting a role of pericytes in regulating coronary microvascular function 79. Deficiency in pericytes results in perturbed vascular development and the formation of microaneurysms in heart 80. During MI, a variety of evidence indicates that dysregulated cardiac pericytes may lead to adverse effects via interference with myocardial blood flow. Abnormal pericytes contraction causes stenosis of cardiac valves and no-reflow in capillaries 81, 82, resulting in decreased coronary blood flow, which may be reversed by administration of the pericyte relaxant adenosine 81. In addition to vasoconstriction, pericytes is also involved in the post-infarction inflammatory response through distinct mechanisms including 1) suppression of inflammation response through releasing the inflammation inhibitory factors such as leukemia inhibitory factor (LIF), cyclooxygenase-2 (COX-2), and heme oxygenase-1 (HMOX-1) 83; 2) attenuation of leukocytes recruitment and transendothelial trafficking through release of chemokines 84; 3) inhibition of the secretion of ECM and proliferation of fibrotic cells through production of matrix metalloproteinases 85; and 4) interference with inflammatory activities of ECs 86.

Given their roles in angiogenesis, coronary microcirculation regulation, and anti-inflammation property, pericytes are also perceived as a promising avenue to foster the repair of microvasculature in infarcted heart. Intramyocardial administration of pericytes is capable of delaying or reversing unfavorable remodeling of infarcted heart through promotion of angiogenesis and inhibition of ECs apoptosis 87. In line with this finding, Katare and colleagues further noted that engraftment of saphenous vein-derived pericytes into infarcted heart drastically improved systolic dysfunction through augmentation of the secretion of angiogenic factors such as VEGF and angiopoietin-1 88. In addition to the soluble cytokines, microRNAs such as miR-132, derived from pericytes after transplantation into the infarcted heart, also restored cardiac function and increased the density of capillary 88. Recently, it was reported that the crosstalk between pericytes and ECs may serve as a necessary bridge for early vasculogenesis. Pericytes are involved in the ECs recruitment, differentiation, maturation and vessel stability through various mechanism including PDGF-B/PDGFRB2, S1P/EDG-1, ANG1/2/TIE2, Cadherin, and Notch signals 89, 90. Nonetheless, further studies are warranted to further elucidate the complex impact of pericyte-endothelial cross-talk in the regulation of coronary microvascular damage and repair following MI.

## Role of MQC in coronary microvascular ECs injury during MI

### Mitochondrial dynamics

**Figure 4:**
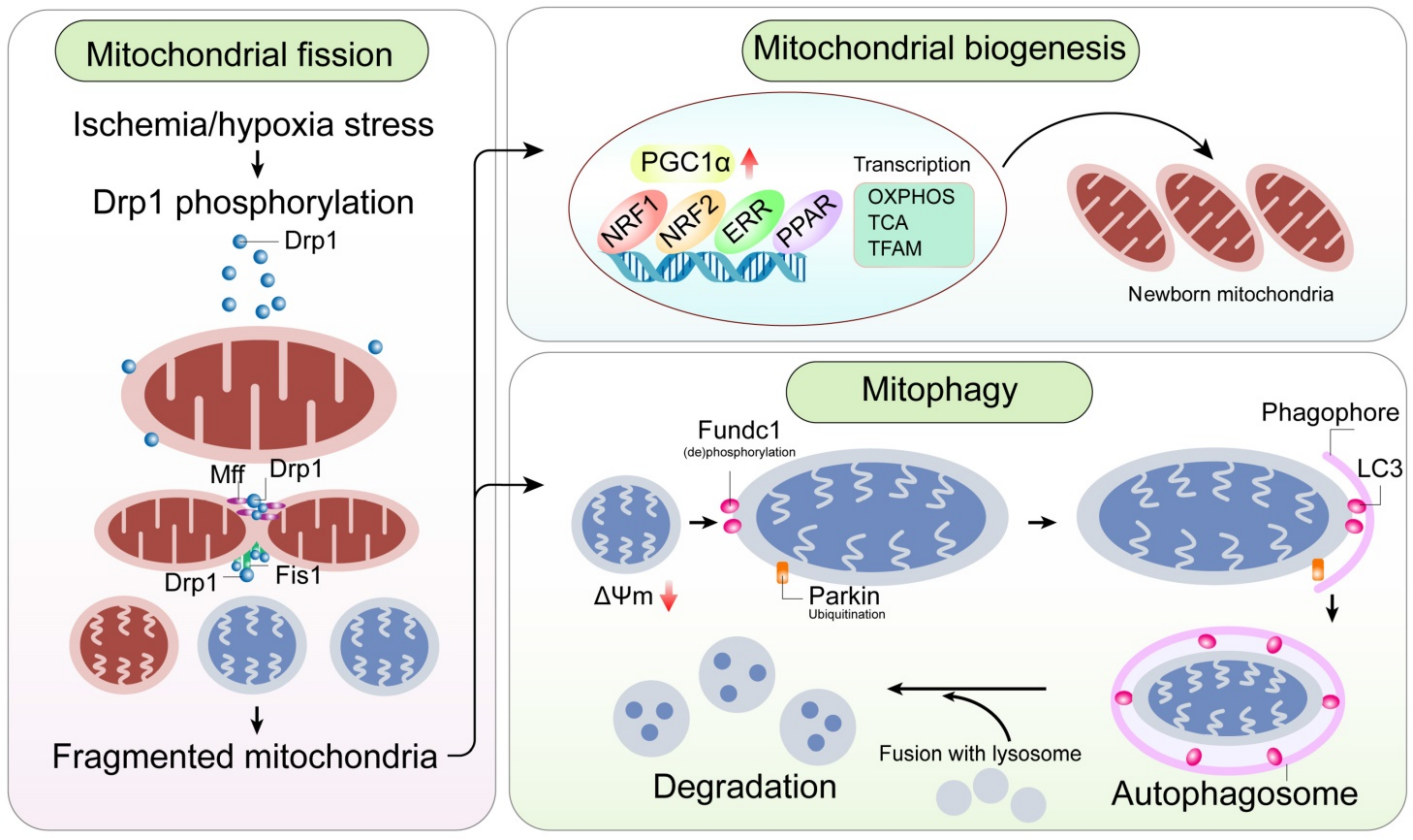
** A mitochondrial-centric view of coronary endothelial homeostasis.** Ischemia/hypoxia promotes enhanced phosphorylation of Drp1. Subsequently, phosphorylated Drp1 (p-Drp1) oligomerizes around the mitochondrial outer membrane after being recruited and stabilized by mitochondrial receptors such as Fis1 and Mff and induces mitochondrial fission. In general, enhanced p-Drp1-mediated mitochondrial fission is accompanied by increased mitophagy and mitochondrial biogenesis. Fragmented mitochondria with low membrane potential induce PINK1 localization into the mitochondria. PINK1 recruits Parkin to the mitochondria. Concurrently, Fundc1, the mitochondria-localized mitophagy receptor, is activated through post-transcriptional phosphorylation. Mitochondria interact with LC3 on the lysosomes through Parkin and Fundc1, and form autophagosomes. Mitophagy promotes degradation of fragmented mitochondria to sustain mitochondrial homeostasis. Mitochondrial biogenesis is activated in response to mitochondrial fission or mitophagy. PGC1α is a major regulator of mitochondrial biogenesis that acts as a compensatory mechanism to mitochondrial fission or mitophagy. The other transcription factors involved in mitochondrial biogenesis are NRF1, NRF2, ERR, and PPAR. Mitochondrial biogenesis upregulates OXPHOS, TCA cycle, mtDNA and TFAM levels. Note: Drp1, dynamin-related protein 1; Fis1, mitochondrial fission factor 1; Mff, mitochondrial fission factor; NRF, nuclear respiratory factor; OXPHOS, oxidative phosphorylation; PGC1α, peroxisome proliferator-activated receptor g coactivator 1α; PINK1, phosphatase and tensin homolog-induced putative kinase 1; ROS, reactive oxygen species; TCA, tricarboxylic acid; TFAM, mitochondrial transcription factor A.

Mitochondria are highly dynamic cellular organelles that continuously undergo fusion (two individual mitochondria join together to become one) and fission (one mitochondrion divides into two) **(Figure 4)**. In ECs, mitochondria exist as filamentous networks under normal physiological conditions and are fragmented during hypoxia 91.

Mitochondrial fission is a multi-step process that involves constriction and scission of the mitochondrial inner membrane through a process mediated by dynamin-related protein 1 (Drp1) and its binding partners, including fission protein 1 (Fis1), mitochondrial fission factor (Mff), mitochondrial dynamics protein of 49 kDa (MiD49), and mitochondrial dynamics protein of 51 kDa (MiD51) **(Table 1)**. Drp1 is a cytoplasmic protein lacking a membrane-anchoring domain. It assembles and oligomerizes into rings which wrap around mitochondria, in concert with its binding partners promotes mitochondrial fission. During normal physiological conditions, mitochondrial fission coordinates proliferation of ECs so that adequate number of mitochondria are received by daughter cells. However, pathological mitochondrial fission may turn on unfavorable mitochondrial apoptotic pathway in the ECs.

Mitochondrial fusion is controlled by mitofusin proteins (Mfn1/2) and optic atrophy 1 (OPA1), which are located on the outer mitochondrial membrane (OMM) and inner mitochondrial membrane (IMM), respectively** (Table 1)**. Mfn1 and Mfn2 form homodimers or heterodimers to induce OMM fusion, whereas, integration of IMM is controlled by OPA1. Fusion of two mitochondria would promote the mixture of mitochondrial contents including mitochondrial DNA, metabolites, and mitochondria-resident proteins, and is deemed a necessary step to remodel structural organization and restore functional capacity of mitochondria for damaged DNA, membranes, or proteins.

#### Mitochondrial fission

Endothelial cell senescence and dysfunction in high glucose conditions is associated with increased mitochondrial fission 92. Mouse coronary ECs from diabetic mice exhibit elevated ROS levels, fragmented and shorter mitochondria, and increased cell death when grown in high glucose (hyperglycemic) conditions 92. In a mouse model of type 1 diabetes, mitochondria show fragmented morphology in the coronary ECs because of increased expression of Drp1 and decreased levels of OPA1^93^. Shenouda and colleagues noted increased mitochondrial fragmentation, mitochondrial ROS levels, and Fis1 expression in the venous ECs isolated from diabetic patients compared to those from healthy subjects 94. Furthermore, siRNA-mediated knockdown of Fis1 or Drp1 inhibited mitochondrial fragmentation and impaired production of NO in the ECs 94. This suggested that mitochondrial fission played a central role in EC dysfunction. Diabetes is an independent risk factor for the onset and development of atherosclerosis and MI. Anti-diabetic drugs such as metformin alleviate diabetes-mediated atherosclerosis through inhibition of Drp1-dependent mitochondrial fission, subsequently improving EC function and reducing vascular inflammation 95. Moreover, human endothelial cells (HUVEC) treated with a single dose of hydrogen peroxide to induce oxidative stress demonstrate transient changes in mitochondrial morphology and mtDNA damage 96.

In post-ischemic or reperfused hearts, increased Mff protein expression and phosphorylation induces mitochondrial fission 97-99. Following ischemia/reperfusion, levels of dual-specificity protein phosphatase 1 (DUSP1) are significantly downregulated in coronary ECs, thus activating JNK, prior to translocation to the nucleus to promote Mff transcription 99. During post-ischemic reperfusion, nuclear receptor subfamily 4 group A member 1 (NR4A1) is upregulated and the serine/threonine kinase, casein kinase2 α (CK2α), is activated in coronary ECs 98. Subsequently, CK2α phosphorylates Mff at Ser-146 to promote its affinity to Drp1. Transcriptional modification and post-transcriptional phosphorylation of Mff enhances Drp1 recruitment onto the surface of mitochondria, leading to excessive mitochondrial fission in coronary ECs during myocardial ischemia/reperfusion injury. In a model of renal ischemia/reperfusion injury, RNA-binding protein Pumilio2 (Pum2) was significantly downregulated in renal tubular cells, thus enhancing the stability and translation of Mff mRNAs 100. However, this novel regulatory mechanism of Mff remains to be examined in coronary ECs.

Mechanical forces also participate in the regulation of mitochondrial dynamics. Breton-Romero and associates demonstrated that alteration in shear stress, a known inducer of atherosclerotic plaque formation or rupture, may promote Drp1 translocation from the cytosol to the mitochondrial membranes to favor mitochondrial fission in coronary ECs 101. This finding suggests that mitochondria respond to signals transduced by changes in fluidic mechanical forces of shear stress in the vascular bed through induction of mitochondrial fission. Along the same line, Chen *et al* noted that mitochondrial fission inhibitor, mdivi-1, suppressed endothelin-1-induced vasoconstriction 102.

Microvascular outgrowth by enhancing myocardial blood perfusion is a protective mechanism against MI. However, angiogenic property of coronary ECs is blunted by myocardial ischemia/reperfusion through complex mechanisms. Treatment with mdivi-1 (an inhibitor of mitochondrial fission) prior to myocardial ischemia/reperfusion challenge ameliorates cardiac anomaly and promotes angiogenesis by upregulating the gap junction protein connexin 43, chemotactic factor CXCL10, and endothelial cell-specific receptor tyrosine kinase 103. Reminiscent to Mdivi-1, calpain inhibition is able to prevent hyperglycaemia-mediated mitochondrial fission while restoring mitophagy to prevent simulated ischemia-reperfusion injury 104. Increased mitochondrial fission correlates with mtDNA damage and reduced proliferative capacity of the ECs, which may be related to impaired MI-related angiogenesis 96. These findings suggest that inhibition of mitochondrial fission may serve as a potential therapeutic avenue for patients with chronic MI through augmentation of angiogenesis.

Besides, it was reported that other mitochondrial functions, such as respiration, ATP formation, and control of calcium kinetics may also be affected by myocardial ischemia/reperfusion injury prior to mitochondrial fission induction 105, suggesting possible mitochondrial function adaptions independent of MQC in myocardial ischemia/reperfusion injury. Therefore, it is necessary to point out that assessment of mitochondrial dysfunction and MQC in MI is rather complex due to the existence of various cross talk machineries.

#### Mitochondrial fusion

Compared with mitochondrial fission, mitochondrial fusion is not well studied in dysfunctional coronary ECs during MI. Makino *et al* reported that downregulation of OPA1 expression promoted mitochondrial fragmentation in coronary ECs isolated from diabetic mice 93. Furthermore, high-fat diet (HFD) significantly induced atherosclerotic phenotype in apoE-deficient mice and downregulated Mfn2 and OPA1 106. Conversely, a fish oil enriched diet retarded the progression of atherosclerotic lesions through upregulation of Mfn2 and OPA1 and endothelium-dependent vasorelaxation response to acetylcholine 106. These observations suggested that mitochondrial fusion prevented formation of coronary atherosclerotic lesions.

Mfn1/2 regulate mitochondrial fusion and VEGF-mediated angiogenic function in coronary ECs 107. Jesse and team reported that VEGF overtly upregulated Mfn2 and Mfn1 levels in cultured ECs 107. Mfn1/2 silencing increased mitochondrial fragmentation and disrupted VEGF-mediated migration and differentiation of ECs* in vitro* 107. It was also reported that reduced mitochondrial fusion contributes to oxidative stress and apoptosis in ECs 108.

Downregulation of mitochondrial fusion proteins under stress has been reported in a number of clinical studies. For example, Diaz-Morales *et al* reported that mitochondrial fusion-related proteins such as Mfn1/2 and OPA1 were significantly downregulated in circulating ECs or EPCs isolated from diabetic patients compared to healthy volunteers 109. Furthermore, reduced mitochondrial fusion and increased mitochondrial fission in ECs of diabetic patients correlated with elevated leukocyte-EC interactions, indicating higher degree of vascular inflammation 109. These findings would suggest that extracellular vascular inflammation is regulated by mitochondrial fusion status in leukocytes and other cell types.

### Mitophagy

#### Mechanism of mitophagy

Mitochondrial fission and fusion are self-regulatory mechanisms in response to stress. However, when damaged mitochondria cannot be repaired by fission or fusion, mitochondrial autophagy (mitophagy) is activated to remove damaged mitochondria in conjunction with autophagolysosomes **(Figure 4)**. At the molecular level, damaged mitochondria are marked by several “adaptors” that are recognized and ingested by lysosomes and hydrolyzed into amino acids, glucose, fatty acids, and nucleotides, which are recycled to synthesize new mitochondrial and other cellular components. Thus, mitophagy requires specific and well-orchestrated communication between mitochondria and lysosomes, and serves as a compensatory mechanism to maintain the quality and quantity of mitochondrial network in cells.

Parkin and FUN14 domain containing 1 (Fundc1) are two specific mitophagic adaptors** (Table 2)**. Parkin induces mitochondrial receptor-independent mitophagy, whereas, Fundc1 mediates mitochondrial receptor-dependent mitophagy 110. Collapse in mitochondrial membrane potential and activity of presenilin-associated rhomboid-like (PARL) proteases in damaged mitochondria promotes accumulation of PTEN-induced kinase 1 (PINK1) on the outer mitochondrial membrane (OMM) and recruitment of cytoplasmic Parkin onto mitochondria via PINK1. Then, Parkin polyubiquitinates several OMM proteins such as voltage dependent-anion-selective channel 1 (VDAC1) and Mfn1/2; the polyubiquitinated proteins are recognized by p62, which interacts with light chain 3 (LC3) to form autophagosomes. Fundc1 is an OMM protein that interacts with LC3 when dephosphorylated and induces mitophagy 111.

#### Mitophagy and EC protection

A vital role of mitophagy is widely reported in endothelial protection. Mitochondrial energy metabolism and endothelial function are maintained by mitophagy through a mechanism involving activation of the Sirt1/FOXO3 signaling cascade 112. Oxidized LDL promotes EC injury, characterized by decreased mobilization, impaired vessel sprouting and blunted tube formation, although these effects are partially reversed by mitophagy induction 113. Mitophagy was shown to retard EC senescence in the settings of hyperglycemia or hyperlipidemia by way of reduced mROS production 114. Inhibition of mitophagy by Parkin silencing overtly lowered mitochondrial membrane potential, induced apoptosis and promoted mitochondrial fragmentation in ECs 115. This finding favors a possible compensatory role for mitophagy in the face of deranged mitochondrial dynamics and mitochondrial fragmentation.

Angiogenesis is vital for cardiomyocyte perfusion and metabolism in the setting of MI. Mitophagy induction correlates with elevated levels of CD31 and VEGF 98. This notion suggests that angiogenesis is positively regulated by mitophagy. Meanwhile, mitophagy protects against mitochondria damage-induced apoptosis of ECs 98. Moreover, mitophagy induction promotes improved vascular relaxation, although the underlying molecular basis remains elusive. Another cardinal feature in MI-associated cardiac remodeling is interstitial fibrosis, which involves vascular matrix remodeling and inflammation. Mitophagy prevents activation of matrix metalloproteinases (MMPs) within the mitochondria and cytoplasm, thereby reducing MMP levels in the extracellular matrix 116. ECs from infarcted cardiac tissues exhibited significantly higher surface levels of adhesive factors such as ICAM1 or VCAM1, the effect of which was reversed by induction of mitophagy 98. In MI, coronary ECs present increased expression of pro-inflammatory factors such as IL-8, MMP9, and MCP1, the effects of which were also reversed by mitophagy 98. To this end, it may be concluded an anti-inflammatory role for mitophagy in coronary ECs during MI.

In MI, mitophagy is induced upon exposure to hypoxia and acts as a hypoxic pre-conditioning mechanism. After exposure to hypoxia, mitochondrial membrane potential is reduced and Parkin translocates to the mitochondria in ECs to trigger induction of mitophagy 117. However, reoxygenation following hypoxia suppresses mitophagy, which causes accumulation of damaged mitochondria that mediate mitochondrial apoptosis in coronary ECs 98, 118 and cardiomyocytes 119. In sum, it is generally accepted that ischemia triggers mitophagy to attenuate mitochondrial damage, whereas, post-ischemic reperfusion inhibits mitophagy. However, regardless of ischemia or reperfusion, suppression of mitophagy induces mitochondrial dysfunction and cell death. This suggests that baseline mitophagy is indispensable for mitochondrial function and cell viability.

### Mitochondrial biogenesis

Mild mitochondrial stress is rectified by mitochondrial fission or fusion, whereas severely damaged mitochondria are removed by mitophagy, resulting in significant reduction in mitochondrial mass **(Figure 4)**. Therefore, de novo mitochondrial biogenesis is required to maintain adequate mitochondrial mass and function. Mitochondrial biogenesis involves transcription and assembly of over 1100 proteins encoded by both nuclear and mitochondrial DNA in addition to synthesis of phospholipids to generate the inner and outer mitochondrial membranes. Peroxisome proliferator activated receptor γ co-activator (PGC1α) and nuclear respiratory factor 2 (NRF2) act as transcriptional factors of most mitochondrial proteins and are considered as master regulators of mitochondrial biogenesis. Mechanistically, PGC1α activates NRF2 and promotes synthesis of nuclear-encoded mitochondrial-resident proteins including mitochondrial transcription factor A (TFAM). Level of PGC1α is tightly regulated by physiological cues including exercise, cold temperature, and fasting 120. PGC1α-mediated mitochondrial biogenesis has been widely described in endothelial protection. Global *Pgc1α* knockout in mice displayed smaller arteries with deformed vascular plexus, low perfusion feature and defective adherent junctions between ECs due to lower levels of membrane-bound VE-cadherin 121. Moreover, *Pgc1α* knockout ECs exhibited a pre-matured aging phenotype with elevated ROS levels 121. In *Pgc1α*-knockout mice, endothelium-dependent vasodilation was suppressed due to reduced NO and ATP levels as well as delayed mitochondrial turnover rate 122. Compared with wild-type mice, *Pgc1α*-knockout mice are more likely to develop atherosclerosis due to impaired lipid metabolism and defective mitochondrial biogenesis 123.

Under physiological conditions, PGC1α enhances resistance to oxidative stress by inducing hemeoxygenase-1 in ECs 124. Exercise-induced angiogenesis is attributed to higher PGC1α expression, which enhances mitochondrial function and promotes paracrine function of VEGF including increased proliferative capacity of ECs 125. PGC1α also promotes viability of ECs and vascular permeability 126. Decreased PGC1α is associated with increased levels of pro-inflammatory factors including TNFα, NF-κB, MCP1 and VCAM1 in ECs 127. Conversely, adenovirus-mediated overexpression of PGC1α reduces ROS production and suppression of NF-κB, MCP1 and VCAM1 triggered by TNFα 127. These findings strongly favor an anti-inflammatory role for PGC1α in vascular homeostasis.

PGC1α is a repressor for oxidative stress, hyperglycemia, hyperlipidemia, and inflammation, all of which are risk factors for MI. In diabetic rats, level of PGC1α is significantly reduced in association with low capillary volume 128. Low-intensity running was shown to enhance capillary volume and pro-angiogenic factors by augmenting PGC1α levels, thereby combating microcirculatory complications in type 2 diabetes 128. PGC1α-related mitochondrial biogenesis may be credited for several anti-diabetic drugs such as thiazolidinediones 129. Supplementation of thiazolidinediones attenuated hyperglycemia-induced EC dysfunction, although such these effects were abrogated in *Pgc1α*-deficient ECs 129. Vascular inflammation induced by angiotensin II was also shown to be associated with reduced PGC1α levels 130. In comparison with healthy individuals, patients with pathological left ventricular outflow tract, circulating PGC1α levels were overtly reduced correlating with increased metabolic biomarkers for mitochondrial damage (lactate, ratio lactate/pyruvate) and EC injury (asymmetric dimethylarginine and total homocysteine) 131.

A recent study (*in vivo* and *in vitro*) showed that thrombospondin mediates Drp1 signaling after ischemia/reperfusion in aged hearts. Using RAEC, an association was shown between thrombospondin and Drp1 through PGC1a 132. In post-ischemic heart, loss of PGC1α levels facilitated mitochondrial fragmentation and promotes cardiac dysfunction 132. This suggests that PGC1α-related mitochondrial biogenesis can potentially alleviate pathogenesis underlying mitochondrial dynamics-related disorders. The role for ROS generation in ischemia/reperfusion was demonstrated by the finding with treatment of mitochondria-targeted antioxidants with improved PGC1α-related mitochondrial biogenesis, mitochondrial respiration, endothelium-dependent vasodilation, and blood flow in ischemic hind limbs 133. PGC1α expression also correlates with reduced angiogenesis in infarcted hearts and pressure overload-induced cardiac hypertrophy 134. PGC1α preserves angiogenesis but not mitochondrial oxidative phosphorylation and contractile function in mice following transverse aortic constriction 134.

Taken together, PGC1α-mediated mitochondrial biogenesis plays a key role in vascular integrity through regeneration of new mitochondria to replenish aged or damaged mitochondria in the governance of mitochondrial quality control. Nonetheless, clinical trials targeting PGC1α-related mitochondrial biogenesis have not been successful thus far in patients with MI.

## Cardioprotective strategies on coronary microvascular injury during MI

### Ischemic preconditioning (IPC)

IPC refers to the ability of brief episodes of ischemia to improve the resistance of myocardium to subsequent ischemic attack. Ample evidence has gained to ensure the protective of IPC in ischemic myocardium. More importantly, IPC also benefits coronary vasculature, as evidenced by preserved endothelial function and decreased ECs damage 135. With the help of IPC, flow-mediated coronary dilator response to vasodilators such as adenosine and NO was also restored to near-normal levels 136. Post-infarction inflammation response, including increased neutrophil adherence and accumulation 136, was also attenuated by IPC, possibly due to improved ECs integrity. The protective mechanisms of IPC have been widely documented in experimental models of myocardial ischemia/reperfusion injury. IPC contributes to activation of K_ATP_ channels, leading to hyperpolarization of ECs 137. This alteration widens the electrochemical gradient to facilitate calcium entry, leading to augmented release of NO from ECs 138. Moreover, IPC treatment is also tied with adenosine release and activation of adenosine receptors 139, resulting in activation of K_ATP_ channels via a G protein-dependent mechanism and thus microcirculation dilation. Besides, IPC suppresses abnormal ROS production 140 and augments the release of prostaglandin E2 141. Levels of endothelin-1 142 are inhibited whereas the content of eNOS is increased 143 by IPC in coronary microvasculatures.

### Ischemic postconditioning (IPost)

IPost refers to a series of brief ischemia/reperfusion cycles applied immediately to the site of ischemic organ following reperfusion, resulting in decreased MI size and improved cardiac function. Intensive effort has been initiated to evaluate the therapeutic potential of IPost for its protection of coronary vasculature against MI. Microvascular obstruction is alleviated and coronary blood flow is preserved in reperfused hearts following IPost 144. It is necessary to point out that several studies failed to display any beneficial actions of IPost on microvascular obstruction 145-147. In addition, one study 145 did not note any reduction in MI size with IPost whereas the others 146, 147 observed a smaller MI zone with the IPost procedure. Although these conflicting findings are difficult to be explained at this time, the mechanism for IPost in coronary microvascular protection has been widely discussed. Decreased myocardial oedema and attenuated neutrophil adherence have been observed in reperfused hearts tissue after treatment with IPost 148. Besides, levels of endothelial P-selectin are downregulated in response to IPost 148. The mobilization of endothelial progenitor cells is also improved by IPost 149, an endogenous repair mechanism for re-endothelization and neoangiogenesis.

### Pharmacological strategies

Vast efforts have been continuously engaged over the past decades in the search for pharmacological agents in the preservation of coronary vasculature against myocardial infarction. For example, angiopoietin-like peptide 4, a hypoxia-dependent factor, plays an important role in regulating vascular permeability. Administration of angiopoietin-like peptide 4 significantly reduces MI size and attenuates microvascular obstruction through regulation of endothelial gap-junction VE-cadherin complex 150. Excessive opening of mitochondrial permeability transition pore (mPTP) has been identified as one of the key pathological mechanisms for myocardial ischemia/reperfusion injury. Cyclosporine-A (CSA), as inhibitor of mPTP, was found to interrupt reperfusion-mediated cardiomyocyte death and therefore reduce MI size 151. However, this beneficial effect has not been verified by large animal experiments 146 and/or large clinical studies 152 given the complexity of the molecular pore structure. Melatonin, which is originally identified as a regulator of circadian rhythm, has been found to sustain coronary microvascular permeability and augment eNOS levels in reperfused heart tissues 118, 153, resulting in decreased MI size and improved cardiac function. Administration of the sodium-glucose cotransporter 2 inhibitors (SGLT2i), a new class of anti-diabetes drugs, significantly reduce MI size and sustain left ventricular mechanoenergetics 154. Furthermore, levels of eNOS are elevated with improved endothelium-dependent vasodilation in response to SGLT2i therapy, suggesting that cardiomyocytes and coronary ECs may serve as targets for SGLT2i in myocardial infarction 154. Although many pre-clinical data support the cardioprotective actions of these pharmacological agents, the neutral results of clinical trials may dampen the enthusiasm of researchers. The translational issues for cardioprotective agents as adjunct to reperfusion therapy with a main focus on coronary microvasculature has been comprehensively discussed by Heusch and colleagues 11, 155-158. A better understanding of signals and molecular mechanisms that regulate coronary ECs function following MI may contribute to identify novel preventive and therapeutic strategies aimed to reduce myocardial injury.

## Pharmacological interventions targeting MQC to protect against coronary microvascular ECs damage

### Mitochondrial dynamics regulation

In addition to mitochondria-targeted antioxidants, several small molecules have been developed and tested to improve MQC and alleviate EC dysfunction. Angiotensin II-treated ECs showed elongated mitochondrial morphology, stabilized mitochondrial membrane potential, increased eNOS expression, decreased apoptosis, and improved migration when pre-treated with mdivi-1, a selective inhibitor of mitochondrial fission protein Drp-1. Rat thoracic aorta and mesenteric arteries exhibited overtly higher Drp1-mediated mitochondrial fission in the realm of endothelin-1-induced vasoconstriction, the effect of which was reversed by mdivi-1 treatment. Mdivi-1 also suppressed metabolic disorder-associated EC defect by increasing NO bioavailability and improving endothelium-dependent vascular relaxation 159. Atherosclerotic mice treated with mdivi-1 showed increased glucose and decreased fatty acid uptake, reduced mitochondrial fragmentation, and decreased expression of p-Drp1^Ser616^ 160. Moreover, mdivi-1 treatment reduced mitochondrial ROS production and VCAM-1 expression in thoracic aorta from atherosclerotic mice 95. In a mouse model of myocardial ischemia/reperfusion injury, mdivi-1 treatment reduced infarct size, improved cardiac function, and induced angiogenesis 161. In addition, mdivi-1 treatment inhibited reperfusion-mediated reduction in connexin 43 levels, upregulation of matrix metalloproteinase (MMP3) and apoptosis of ECs in the reperfused myocardium 162. Mdivi-1 treatment also favored NO production and viability of cultured ECs subjected to shear stress or hypoxia/reoxygenation injury 163. EC cells treated with mdivi-1 displayed improved mitochondrial dynamics and reduced levels of von Willebrand Factor and gap junction communication 164. This demonstrates that normalization of mitochondrial dynamics is necessary to maintain the vascular barrier and anti-coagulant properties.

In contrast to mitochondrial fission, support evidence for pharmacological approaches targeting mitochondrial fusion is relatively fewer. This is mainly due to the multi-faceted roles of Mfn1/2 in the regulation of mitochondrial fusion, mitophagy, mitochondrial calcium levels, and mitochondria-ER communication. Further investigations are necessary to hunt and identify clinically effective mitochondria fusion-targeted drugs for preservation of EC function in MI patients.

### Mitochondrial biogenesis modification

Regular aerobic exercise promotes mitochondrial biogenesis and is recommended as a healthy lifestyle strategy for reducing MI-related risk factors such as obesity, dyslipidemia and diabetes mellitus. Ample evidence has denoted a critical role for MQC in MI-related ECs changes. For example, exercising rats following MI was shown to overtly promote EC proliferation, VEGF level, angiogenesis, eNOS activity, and endothelium-dependent vasodilation 165. In addition to exercise, mitochondrial biogenesis in ECs may be stimulated by various medications including statins 166, resveratrol 167, liraglutide 168, cannabidiol 169, salidroside 170, and red wine polyphenols 171. Nonetheless, only metformin 172 and resveratrol 173 augment mitochondrial biogenesis in ECs, improved cardiac function and decreased infarct size in MI or myocardial I/R injury models.

### Mitophagy induction

Trehalose is a small molecule isolated from mushrooms and honey that activates autophagy and mitophagy in ECs 174. For example, four weeks of oral trehalose supplementation reversed age-associated decline in NO-mediated endothelium-dependent vasodilation 174, 175. Mechanistically, Parkin and BCL2/adenovirus E1B 19 kDa protein-interacting protein 3 (Bnip3) as well as PGC1α expression levels were significantly increased in the aortic tissues after trehalose treatment 175. Administration of trehalose for four weeks reversed cardiac remodeling and fibrosis in the wild-type MI model mice, but did not show beneficial effects in the Beclin1-knockout MI model mice 176. Moreover, trehalose enhanced VEGF-mediated angiogenesis in the *Mycobacterium marinum*-infected zebrafish 177, but, these effects have not been validated in MI-related ECs.

Another natural activator of autophagy is spermidine, isolated from wheat germ, soy beans, and mushrooms 178. Dietary intake of spermidine is inversely associated with all-cause mortality and MI risk in humans 179. Spermidine improved age-associated decline in aortic mitochondrial respiration and reduced inflammation and atherosclerosis through mitophagy induction in hyperlipidemic mice 180. In addition, ECs isolated from veins of diabetic patients exhibited improved NO signaling and NO-mediated endothelium-dependent vasodilation following spermidine treatment 181. In a rat model of MI, spermidine supplement reversed cardiac dysfunction and inhibited cardiomyocyte apoptosis through mitophagy induction 182. However, further evidence is required to demonstrate the beneficial effects of spermidine on ECs during MI through mitophagy activation.

## Concluding remarks and Perspectives

**Figure 5:**
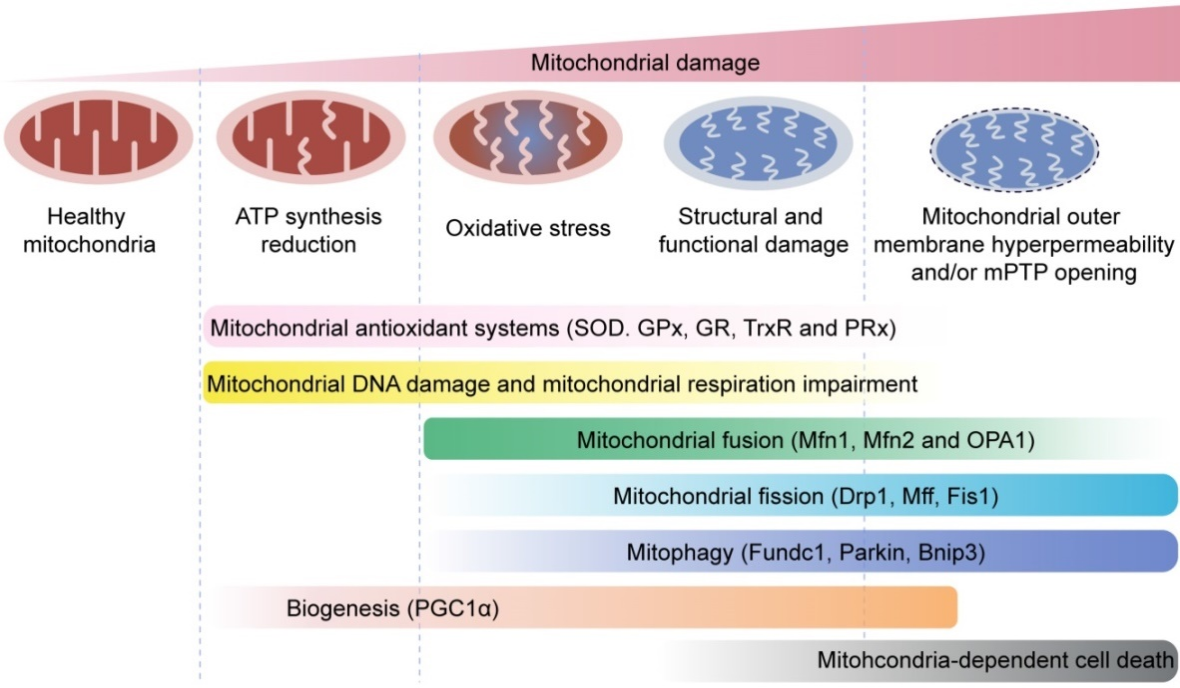
** Mitochondrial quality control mechanisms.** Mild mitochondrial stress is accompanied by decreased ATP production and mitochondrial oxidative stress. This stimulates mitochondrial anti-oxidative enzymes such as superoxidase dismutase (SOD), glutathione reductase (GR), glutathione peroxidases (GPXs), thioredoxin reductase (TrxR), and peroxiredoxin (PRx) to reduce the levels of mitochondrial reactive oxygen species (mROS). Mitochondrial injury is followed by mitochondrial DNA damage, which impairs transcription and translation of mitochondria-encoded electron transport chain proteins. Severe mitochondrial stress is characterized by aberrant alterations in the mitochondrial structure and function. This induces mitochondrial fusion mediated by mitofusin 1 (Mfn1), mitofusin 2 (Mfn2), and optic atrophy 1 (OPA1). Mitochondrial fusion allows mixing of damaged and healthy mitochondria to restore functional homeostasis. Meanwhile, mitochondrial fission is activated by cytosolic dynamin-1-like protein (Drp1) and its receptors, and results in segregation of damaged mitochondria that are then targeted for degradation by mitophagy through specific adaptors such as FUN14 domain-containing 1 (Fundc1), Parkin, and BCL-2/adenovirus E1B 19 kDa protein-interacting protein 3 (Bnip3). Excessive mitochondrial damage and stress promotes increased mitochondrial fission that converts reticular mitochondrial network into punctate and fragmented mitochondria. This reduces ATP production and affects cellular growth and survival. Besides, excessive mitophagy significantly reduces mitochondrial mass leading to bioenergetic crisis and cell death or apoptosis. Newer mitochondria are synthesized by mitochondrial biogenesis through specific transcription factors such as peroxisome proliferator-activated receptor-γ co-activator 1α (PGC1α). However, excessive mitochondrial damage induces cell death.

Coronary microvascular injury is an often neglected topic in the field of MI. Prognosis of cardiovascular disease is worse in women as opposed to age-matched men courtesy of microvascular dysfunction. However, current therapies mainly focused on improving the viability of cardiomyocytes and effective therapies targeting coronary microvascular injury are not yet available. In ECs, mitochondria play a vital role in integrating the intracellular signal transduction pathways that regulate EC function. MQC mechanisms including mitochondrial dynamics, mitophagy and mitochondrial biogenesis play a pivotal role in regulating mitochondrial structure and function. They are also implicated in EC dysfunction and coronary microvascular injury in the infarcted heart tissues **(Figure 5)**. Mitochondrial fusion and fission regulate the number of healthy mitochondria through changes in mitochondrial dynamics. Mitophagy is a self-clearance mechanism that removes damaged mitochondria to maintain mitochondrial function and homeostasis. Mitochondrial biogenesis is the de novo synthesis mechanism to maintain optimal mitochondrial mass, especially following reduced mitochondrial mass due to mitophagy. These three MQC mechanisms regulate mitochondrial function in the ECs. Dysregulated MQC induces oxidative stress in ECs due to excessive accumulation of damaged mitochondria followed by activation of an inflammatory response in cardiac microvasculature. Defective ECs and inflamed vascular bed lower the threshold for vasospasms and facilitate thrombogenesis, thus limiting blood flow in the myocardium during MI. Moreover, dysfunctional ECs undergo apoptosis and fibrosis because of impaired MQC. This is associated with microvascular degeneration, chronic myocardial ischemia, and negative cardiac remodeling during MI. A number of unanswered questions remain with regards to MQC in coronary microvascular injury during MI. First, it is of interest to explore whether the location of ECs (i.e., cells located at the infarcted area versus border zone) possesses any distinct role in regulating microvascular damage during or after MI. Second, it remains unclear whether MQC could be regulated by a common upstream regulator or signaling pathway in ECs. Third, it is of interest to describe the interactive actions between MQC processes. Fourth, although several drugs have been developed or validated for the treatment of coronary microvascular injury, no single drug or therapeutic approach targeting global MQC has yet been proven effective for the protection of coronary microvascular injury.

Last but not the least, this review mainly discusses and summarizes the role of coronary ECs in coronary microvascular damage in myocardial infarction. It is noteworthy to point out that coronary microcirculation, including small arteries and arterioles, is composed of vascular smooth muscle and pericytes, in addition to ECs. Although the direct contact of ECs with blood flow means that they are particularly vulnerable to the damaging molecules in the blood, possible influence from dysregulated vascular smooth muscle cells in coronary microvascular homeostasis cannot be neglected. For example, eNOS-mediated vasodilation response is initiated by ECs and executed by vascular smooth muscle cells. In addition, coronary artery spasm has been reported to interplay between endothelial dysfunction and vascular smooth muscle cell hyperreactivity 183. It requires further investigation and summarization to obtain more complete elucidation of the role vascular smooth muscle cells in MI.

## Figures and Tables

**Table 1:** Post-translational modifications of mammalian mitochondrial dynamics-related proteins

Protein substrate	Regulators	Full name	Post-transcriptional modification	Effects of the modifications	References
Drp1	CDK1	Cyclin-dependent kinase 1	Phosphorylation of Ser 616	Activation	184
	PKA	Protein kinase A	Phosphorylation of Ser637	Inactivation	185
	DNA-PKcs	DNA-dependent protein kinase, catalytic subunit	Phosphorylation of Ser616	Activation	186
	O-GlcNAc transferase or OGT	uridine diphospho-N-acetylglucosamine:polypeptide β-N-acetylglucosaminyltransferase	O-GlcNAcylation of Drp1 at Thr585/586	Activation	187
	MAPL	Mitochondria-associated protein ligase	SUMOylation of Drp1	Activation	188
	SENP5	SUMO-specific Peptidase 5	De-SUMOylation of Drp1	Inactivation/stabilization	189
	March5	Membrane associated RING finger protein 5	Ubiquitination of Drp1	Degradation	190
Mfn1	ERK	Extracellular-signal-regulated kinase 1/2	Phosphorylation of T562	Inactivation	191
	March5	Membrane associated RING finger protein 5	Ubiquitin of Mfn1	Degradation	192
Mfn2	PINK1	PTEN-induced kinase 1	Phosphorylation of Thr111 and Ser442	Signaling	193
	March5	Membrane associated RING finger protein 5	Ubiquitin of Mfn2	Activation	194
Mfn1/2	USP30	Ubiquitin specific peptidase 30	Deubiquitin of Mfn1/2	Inactivation	195
Mff	NR4A1	Nuclear receptor subfamily 4 group A member 1	Phosphorylation of Ser146	Activation	196

**Table 2:** Mitophagy adaptors and their regulatory mechanisms in endothelial cells

Mitophagy Adaptors	Full name	Regulator	Signal	Effects	References
PINK1/Parkin	Phosphatase and tensin homologue (PTEN)-induced putative kinase 1/Parkin	Sarcoplasmic/endoplasmic reticulum Ca^2+^-ATPase (SERCA)	Transcriptional upregulation	Overexpression of SERCA improves endothelial viability, barrier integrity, and cytoskeleton assembly in cardiac microvascular endothelial cells under cardiac ischemia-reperfusion injury by activating Parkin-related mitophagy.	197
		Ca^2+^/calmodulin-dependent protein kinase II (CaMKII)	Post-transcriptional phosphorylated activation	CaMKII induces loss of mitochondrial potential and energy disorder in oxidized low-density lipoprotein (ox-LDL)-treated aortic endothelial cells through activation of Parkin-mediated mitophagy.	198
		Uncoupling protein2 (Ucp2)	Transcriptional downregulation	Loss of endothelial Ucp2 leads to excessive PINK1-induced mitophagy, inadequate mitochondrial biosynthesis, and increased apoptosis in endothelium.	199
		Regulator of calcineurin 1-1L (Rcan1-1L)	Transcriptional upregulation	Rcan1-1L overexpression upregulates Parkin-related mitophagy and reverses growth inhibition in hypoxia-treated endothelial cells.	117
Bnip3	BCL2/adenovirus E1B 19 kDa protein-interacting protein 3	5' AMP-activated protein kinase (AMPK)	Transcriptional upregulation	Inhibition of AMPK attenuates the activity of mitochondrial respiration complexes I and III in endothelial cells under oxidized low-density lipoprotein treatment by blunting Bnip3-related mitophagy.	113
Fundc1	FUN14 domain-containing 1	Nuclear receptor subfamily 4 group A member 1 (NR4A1)	Post-transcriptional phosphorylated inactivation	NR4A1 induces inflammation and apoptosis of cardiac microvascular endothelial cells by inhibiting Fundc1-dependent mitophagy in a mouse model of cardiac ischemia-reperfusion injury.	196
